# Biology and dynamics of potential malaria vectors in Southern France

**DOI:** 10.1186/1475-2875-6-18

**Published:** 2007-02-21

**Authors:** Nicolas Ponçon, Céline Toty, Grégory L'Ambert, Gilbert Le Goff, Cécile Brengues, Francis Schaffner, Didier Fontenille

**Affiliations:** 1 Institut de Recherche pour le Développement (IRD), UR016, Caractérisation et Contrôle des Populations de Vecteurs, 911 avenue Agropolis, BP 64501, 34394 Montpellier cedex 5, France; 2 Entente Interdépartementale pour la Démoustication (EID) Méditerranée, 165 avenue Paul-Rimbaud, 34184 Montpellier cedex 4, France; 3 University of Zuerich, Institute of Parasitology, Winterthurerstrasse 266a, CH-8057 Zuerich, Switzerland

## Abstract

**Background:**

Malaria is a former endemic problem in the Camargue, South East France, an area from where very few recent data concerning *Anopheles *are available. A study was undertaken in 2005 to establish potential malaria vector biology and dynamics and evaluate the risk of malaria re-emergence.

**Methods:**

Mosquitoes were collected in two study areas, from March to October 2005, one week every two weeks, using light traps+CO_2_, horse bait traps, human bait catch, and by collecting females in resting sites.

**Results:**

*Anopheles hyrcanus *was the most abundant *Anopheles *species. *Anopheles melanoon *was less abundant, and *Anopheles atroparvus *and *Anopheles algeriensis *were rare. *Anopheles hyrcanus *and *An. melanoon *were present in summer, whereas *An. atroparvus *was present in autumn and winter. A large number of *An. hyrcanus *females was collected on humans, whereas almost exclusively animals attracted *An. melanoon*. Based on an enzyme-linked immunosorbent assay, almost 90% of *An. melanoon *blood meals analysed had been taken on horse or bovine. *Anopheles hyrcanus and An. melanoon *parity rates showed huge variations according to the date and the trapping method.

**Conclusion:**

*Anopheles hyrcanus *seems to be the only *Culicidae *likely to play a role in malaria transmission in the Camargue, as it is abundant and anthropophilic.

## Background

In recent years, several vector-borne diseases have re-emerged and spread in Europe with major health, ecological, socio-economical and political consequences. Most of these outbreaks are linked to global and local changes resulting from climate change, human induced landscape changes or human population activities. Malaria, a former European endemic disease recently struck European boundaries in countries such as Azerbaijan, Georgia and Turkey where it had been eradicated after World War II [1]. Only a few autochthonous cases have been reported recently in Europe, but these events indicate that the malaria situation needs to be re-examined in Europe [1-5].

In metropolitan France, malaria was endemic until the beginning of the 20^th ^century in marshy areas such as the Landes, the Dombes, Brittany, Alsace, the Rhone delta, Roussillon and Corsica [6]. Then, it decreased drastically due to the drying of marshes, growth of livestock, improvement of housing and life conditions and the use of quinine. The last outbreak was observed in Corsica from 1966 to 1972, with about 30 *Plasmodium vivax *cases [7,8]. Malaria disappeared from the Camargue after World War II [9].

Nowadays, all the malaria cases reported in France are only imported cases [10], excepted for three suspected, but not-confirmed, autochthonous cases in 2006 [11](Doudier, unpublished data). In 2004, the total number of imported cases was 6,109 with a predominance of *Plasmodium falciparum *[12]. Between 1977 and 2000, 28 airport malaria cases following infected mosquito importation were recorded [13] and very few cases have been notified as congenital malaria or accidental blood exposure [10].

Thirteen *Anopheles *species have been reported in metropolitan France [14]. Among them, two species were considered to be primary vectors because of their abundance and their potential anthropophily: *Anopheles atroparvus *in continental France and *Anopheles labranchiae *in Corsica.

Despite drying of some marshes and consecutive reducing of mosquito populations during the 20^th ^century, *Anopheles *mosquitoes are still present in France and could be very abundant in some places generating an "anophelism without malaria" situation. However, global and local changes may modify *Anopheles *biological parameters linked to malaria transmission (vector density, contact between humans and vectors, longevity, species). Only few data on anopheline potential vectors have been collected in France since the seventies [15].

An in-depth longitudinal survey was conducted in the Camargue, to assess vector species and distribution and evaluate mosquito vectorial capacity related to human malaria transmission risk. Dynamics, feeding preferences, parity rates and nightly activity were studied for different *Anopheles *species.

## Methods

### Study area

**Figure 1:**
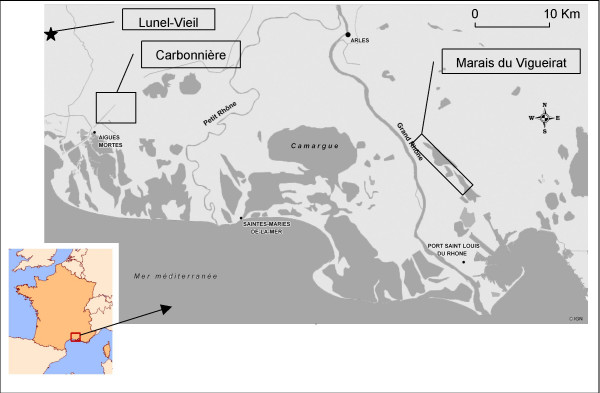
Location of the Camargue and field study areas.

The study was carried out in the Camargue, a large wet area in the South East of France (Figure 1). The main part of the Camargue is located inside the Rhone river delta and it also includes small belts at the east and west sides of the delta. This area has a Mediterranean climate characterized by warm, dry summers and mild, wet winters. Total annual rainfall usually ranges between 500 and 700 mm, with a maximum during October. The annual mean temperature is 14°C. Mean daily minimum and maximum temperatures range from 0°C to 10°C in winter and from 15°C to 30°C in summer (data from "Météo France").

Water pools and marshes cover a large part of its surface. Water is provided either by rains or by a very tight canal network diverted from the Rhone River and used to irrigate paddies or to fill marshes. Management of water is realized individually by field owners depending on use: grazing for horses, cows or sheep, exploitation of reeds or rice and hunting reserves for waterfowl.

As this area is near the sea, it is characterized by a low deep water table presenting a high salinity. Salinity of breeding site water depends on location and artificial or natural submersion frequency. Marsh flora is very dependant on salinity and xerophily. Different vegetation patches are observed in relation to different biotopes and constitute different types of marshes. Moreover, there are various forms of agriculture (including vineyard, paddies, market gardening, fruit growing) and the cultivation of rice is particularly developed and covers more than 18,000 hectares in the Camargue. Livestock includes horses, cows and sheep.

Two study areas were chosen in the Camargue. They are about 45 kilometres apart. The first one, named "Marais du Vigueirat" (4°46'E; 43°30'N) is a natural reserve, where human activity and impact are very limited. Limited visits, reserve maintenance and fauna and flora surveillance are the only human activities. Some horses and cows graze this area. It presents a large variety of biotopes and different types of marshes. The west side is dominated by a large surface of paddy and on its east side by a particular biotope constituted from a resurgence of the water table. Very few people are resident in this area, but a small town named Mas Thibert is located approximately three kilometres away.

The second study area, named "Carbonnière" (4°13'E; 43°35'N), presents the same variety of biotopes and marshes, including paddy. Human presence and activities are more developed: residents, a large number of tourists, camping and hotels, exploitation of wine and reed beds, breeding of horses and cows, and hunting. Moreover, Carbonnière is located in an area of pest control for mosquitoes and in particular against *Aedes (Ochlerotatus) caspius*.

### Mosquito collections

Adult mosquitoes were captured from March to October 2005, one week out of two, in each study area. Specific trapping sessions were conducted until December. The following collection techniques were used:

- Eight CDC-light traps associated with carbon dioxide dry ice were hung in eight locations in each area from 19:00 to 10:00 hours, two consecutive nights, one week in two from March to October. The mean number of mosquitoes collected in each area from eight traps each night was calculated using the results of the two consecutive nights.

- Mosquito activity was recorded by collecting two CDC-light traps+CO_2 _every 2 hours from 20:00 to 08:00 once in August and once in September in each area.

- Hourly human bait collection were made on three adults belonging to the research team from 20:00 to 00:00 and from 02:00 to 04:00, once in August and once in September in each area and from 19:00 to 23:00 in May and from 18:00 to 22:00 in October.

- Three horse bait traps were used at Marais du Vigueirat from 20:00 to 08:00 in May, August and September 2005. The net was hung in a horse shelter near a large opening in the wall. Three others horses were present in the shelter.

- Potential adult resting places were explored regularly from March to December 2005: shelters (with or without animals), bird observatory, medieval tower, water pipe and natural shelters. *Anopheles *females were collected using mouth aspirators. Depending on the observed number of *Anopheles*, the totality or only a fraction was captured. Moreover, collection of mosquitoes in vegetation was realised at the beginning of May using a backpack aspirator.

### Field processing of mosquitoes

*Anopheles *individuals were removed from the rest of the collected mosquitoes. They were identified using morphological characteristics and identification key [16]. Ovaries from a portion of female anophelines captured by light traps were dissected regularly to determine parity [17]. When the number of females caught by light traps was not large enough, females collected in resting sites were also dissected. All mosquitoes from the *Anopheles maculipennis *complex, dissected or not, were stored individually in a numbered tube with desiccant for laboratory processing in Montpellier. Blood meals of blood-fed females were extracted and preserved on filter papers.

### Laboratory processing of anophelines

Females belonging to the *An. maculipennis *complex were identified to species using the PCR technique described by Proft et al. [18]. A leg or a wing was placed directly into the reaction mixture containing the species-specific primers, dNTPs, buffer and polymerase. The length of amplified sequences was 410 nucleotides for *Anopheles maculipennis s.s*., 224 for *Anopheles melanoon *and 114 for *An. atroparvus*. When more then 30 females were collected during two consecutives nights at the same site by the same method, a random sample was selected. The probable number of specimens per species was extrapolated.

Blood meal sources of fed females collected by light traps and in resting places were identified by an enzyme-linked immunosorbent assay (ELISA) [19]. The technique identified human, bovine, sheep or goat, horse or donkey, chicken, pig, rat, dog, guinea pig and rabbit. When too many females were collected in the same resting site for one day, a random sample was processed.

## Results

### Species

From March to October 2005, 131,050 *Anopheles *belonging to *An. maculipennis *complex, *Anopheles hyrcanus *and *Anopheles algeriensis *were collected in both areas. The number of *Anopheles *captured by each method, per study area and per month is reported in Table 1. Of the 5,031 *An. maculipennis *complex specimens, 1,830 were identified by PCR. Two sibling species were reported: *An. melanoon *and *An. atroparvus*.

**Table 1:** Total number of *Anopheles *caught in 2005 by methods and months in the Camargue

Study area	Capture method	Species	Mar.	April	May	June	July	Aug.	Sept.	Oct.	Nov.	Dec.	Total
Carbonnière	Light trap	*An. melanoon*	0	0	10	39	45	122	18	0	-	-	234
		*An. atroparvus*	0	0	0	1	1	1	1	0	-	-	4
		*An. hyrcanus*	0	0	16	673	374	3,257	1,228	3	-	-	5,551
	Resting fauna	*An. melanoon*	0	1	3	114	-	182	76	10	2	0	388
		*An. atroparvus*	0	0	0	7	-	14	8	82	59	4	174
		*An. hyrcanus*	0	0	0	0	-	0	0	0	0	0	0
	Human landing	*An. melanoon*	-	-	0	-	-	0	0	-	-	-	0
		*An. atroparvus*	-	-	0	-	-	0	0	-	-	-	0
		*An. hyrcanus*	-	-	0	-	-	398	35	-	-	-	433
	Total	*An. melanoon*	0	1	13	153	45	304	94	10	2	0	622
		*An. atroparvus*	0	0	0	8	1	15	9	82	59	4	178
		*An. hyrcanus*	0	0	16	673	374	3,655	1,263	3	0	0	5,984

Marais du Vigueirat	Light trap	*An. melanoon*	0	6	27	134	464	1,073	105	2	-	-	1,811
		*An. atroparvus*	0	0	0	0	0	0	0	0	-	-	0
		*An. hyrcanus*	5	7	296	6,737	17,739	61,315	25,708	124	-	-	111,931
		*An. algeriensis*	0	43	50	72	2	0	1	2	-	-	170
	Resting fauna	*An. melanoon*	0	0	146	292	-	361	128	5	0	-	932
		*An. atroparvus*	0	0	5	6	-	6	20	9	6	-	52
		*An. hyrcanus*	10	0	3	0	-	2	0	0	0	-	15
		*An. algeriensis*	-	0	-	0	-	0	0	0	0	-	0
	Horse bait	*An. melanoon*	-	-	1	-	-	1,393	17	-	-	-	1,411
		*An. atroparvus*	-	-	0	-	-	15	0	-	-	-	15
		*An. hyrcanus*	-	-	0	-	-	768	88	-	-	-	856
		*An. algeriensis*	-	-	0	-	-	0	0	-	-	-	0
	Human landing	*An. melanoon*	-	-	0	-	-	10	0	0	-	-	10
		*An. atroparvus*	-	-	0	-	-	0	0	0	-	-	0
		*An. hyrcanus*	-	-	0	-	-	7,034	14	14	-	-	7,062
		*An. algeriensis*	-	-	1	-	-	0	0	0	-	-	1
	Total	*An. melanoon*	0	6	174	426	464	2,837	250	7	0	-	4,164
		*An. atroparvus*	0	0	5	6	0	21	20	9	6	-	67
		*An. hyrcanus*	15	7	299	6,737	17,739	69,119	25,810	138	0	-	119,864
		*An. algeriensis*	0	43	51	72	2	0	1	2	0	-	171

-: Not done

### Population dynamics

**Figure 2:**
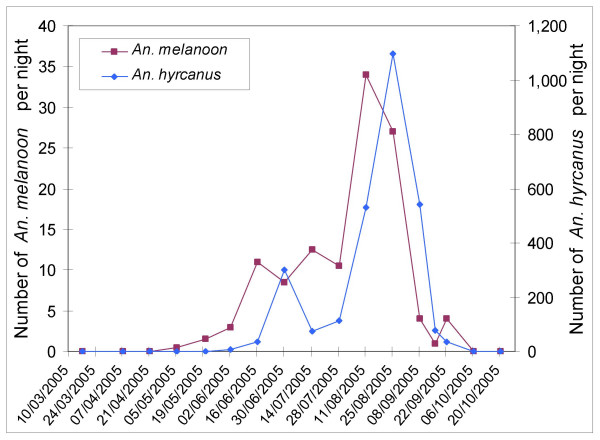
Seasonal dynamics of *Anopheles melanoon *and *Anopheles hyrcanus *at Carbonnière in 2005 (eight traps).

**Figure 3:**
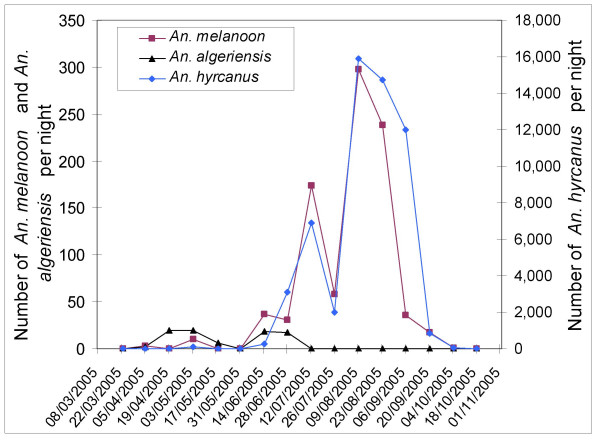
Seasonal dynamics of *Anopheles melanoon*, *Anopheles hyrcanus *and *Anopheles algeriensis *at Marais du Vigueirat in 2005 (eight traps).

Dynamics of *An. hyrcanus*, *An. melanoon *and *An. algeriensis *based on light traps are presented in Figures 2 and 3, for each study area. Results show the mean number of mosquitoes collected in the area from the eight traps per night. The number of *An. atroparvus *collected by light traps or in resting sites was low and mosquitoes were mainly captured between August and November.

### Activity and aggressiveness

*Anopheles hyrcanus *was active at the beginning of the night and presented a very marked peak of biting just after the sunset while *An. melanoon *appeared as active in the second part of the night.

### Resting fauna

Number of mosquitoes collected in resting sites is shown in Table 1. Because sampling effort varied along the season, results do not necessarily reflect dynamics. For both study areas, the maximum *An. melanoon *captured was in August 2005. They were found only in anthropic, dark, calm areas without any signs of draught and with or without animals. They were never captured in vegetation or in natural shelters. During the rest of the season, it was only found in resting sites directly related to animals. *Anopheles atroparvus *was essentially found in horse shelters.

### Feeding preferences

A total of 267 bloodmeals were tested by ELISA. Blood-fed females were captured in resting sites and light traps. Results per species are presented in Table 2.

**Table 2:** Blood meal analysis of fed mosquitoes

			No of mosquito fed on each vertebrate host					
Mosquito species	Study area	No of mosquitoes	Horse	Bovine	Boar	Mixed	Dog	Other hosts

*An. melanoon*	Carbonnière	142	95	41	0	2^a^	0	4
	Marais du Vigueirat	84	67	1	8	0	1	7
*An. atroparvus*	Carbonnière	12	10	2	0	0	0	0
	Marais du Vigueirat	3	3	0	0	0	0	0
*An. maculipennis *complex	Carbonnière	11	9	2	0	0	0	0
	Marais du Vigueirat	6	2	1	2	1^b^	0	0
*An. hyrcanus*	Carbonnière	1	1	0	0	0	0	0
	Marais du Vigueirat	8	7	0	1	0	0	0

Total		267	194	47	11	3	1	11

^a ^: horse/bovine and horse/boar

^b ^: horse/sheep

### Parity rates

Parity rates varied during the season for each species and between species. Results are presented in Table 3. During summer period (June, July and September), parity rates were 0,48 (0,41–0,54) and 0,46 (0,27–0,66) for *An. hyrcanus *and *An. melanoon *respectively at Carbonnière and 0,32 (0,27–0,38) and 0,54 (0,43–0,65) for *An. hyrcanus *and *An. melanoon *respectively at Marais du Vigueirat.

**Table 3:** Parity rates of *Anopheles *captured in 2005

	Carbonnière									Marais du Vigueirat					
	*An. melanoon*			*An. atroparvus*			*An. hyrcanus*			*An. melanoon*			*An. hyrcanus*		

	Capture	P	No	Capture	P	No	Capture	P	No	Capture	P	No	Capture	P	No

Mar.	-	-	-	-	-	-	-	-	-	-	-	-	LT+RF	0.71	7
April	-	-	-	-	-	-	-	-	-	LT	0.75	4	LT+human	0.80	10
June	LT	0.40	5	-	-	-	LT	0.14	7	RF	0.07	30	LT	0.68	25
July	LT	0.50	14	-	-	-	LT	0.22	87	LT	0.29	48	LT	0.24	118
Sept.	LT	0.44	9	LT	0.00	1	LT	0.67	132	LT	0.91	33	LT	0.33	118
	RF	0.50	32	RF	1.00	4	-	-	-	RF	0.26	19	-	-	-
Oct.	RF	0.14	7	RF	0.00	48	-	-	-	-	-	-	-	-	-
Dec.	-	-	-	RF	0.00	3	-	-	-	-	-	-	-	-	-

P: Percentage of parous females

No: Number of dissected females

LT: Light trap

RF: Resting fauna

-: Not done

## Discussion

*Anopheles hyrcanus *was the most collected species in both areas and particularly at Marais du Vigueirat where thousands of specimens were captured per night during the peak. Rice fields constitute prolific larval sites for this species and large populations are frequently associated with some rice growing areas as described in Turkey or Greece [20]. Although it was much less abundant than *An. hyrcanus*, *An. melanoon *was predominant within the Maculipennis complex in the two study areas. Rioux noticed this abundance by the years 1950: *An. melanoon *was very abundant in the coastal area and paddies constituted its favourite breeding site [21]. Salières confirmed these observations 20 years later [15]. Data presented here show that *An. melanoon *abundance has not diminished in the Camargue from the years 1950. *Anopheles atroparvus *was very rare in 2005, although it had been described as the major vector in the past. It was very abundant and present in several environmental patterns of South East France by the 1940's and 1950's [9,21], whereas Salières captured it only five times during a 4-year survey 20 years later [15]. Observations reported here confirmed that this species remained very rare.

Breeding sites of *An. algeriensis *are constituted by resurgence of water tables [21]. This kind of biotope is located on the east side of the Marais du Vigueirat where the "Crau" water table appears. This is why this species was captured only at Marais du Vigueirat and particularly in spring. The absence of *An. algeriensis *in summer could be explained by resurgences being dry during summer due to drought in the South of France.

*Anopheles maculipennis s.s*. was reported several times in France, but it is associated with fresh water [22]. It was reported only twice near the south coast of France and in fresh water biotopes [15,21]. Moreover, 16 *An. maculipennis *complex specimens were collected in a horse bait trap in 2004 at Lunel-Viel [23]. Six out of 11 specimens processed by PCR were identified as *An. maculipennis s.s*. Lunel-Viel is located at the outer limits of the Camargue, in a dry area without any marshes, paddies or pools submitted to salt water table resurgence. The absence of *An. maculipennis s.s*. in results presented here is in accordance to these environmental differences.

*Anopheles claviger s.s*., *Anopheles petragnani *and *Anopheles plumbeus *have been reported several times in south-east France. They were not captured during the survey conducted in 2005 because their ecological niches were rare or absent of the two sites and this study did not focus on their breeding sites (which correspond to tree holes for *An. plumbeus*, rivers stream and small, cold and fresh water collection for the others) [14,21,24,25]. However, *An. plumbeus *and *An. claviger*, which were considered as secondary vectors, could be more abundant in some others places, and particularly places close to humans.

*Anopheles labranchiae *has not been recorded in the Camargue and continental France so far, while it is abundant in Corsica, where it is reported in breeding sites such as small pools with fresh water and marshes [14,26,27]. It is also abundant in Italy, particularly in paddies and in rivers and streams [28]. In the context of global warming, distribution of this species has not yet expanded to the Camargue despite abundance of potential breeding sites, such as paddies.

*Anopheles hyrcanus *and *An. melanoon *presented similar dynamics in the two areas in 2005, although total mosquito numbers were very different. Their populations began increasing in the middle of June, reaching a peak near the middle of August and decreasing drastically in the middle of September (although *An. melanoon *decreased earlier). Dynamics of these species collapsed brutally at the end of July in the two areas without any identified cause (such as wind, temperature, treatment by mosquito pesticide, hygrometry, water supplying of breeding sites, significant modification of *Anopheles *breeding sites or moon cycle).

Populations of resting *An. atroparvus *remained very low until September or October and then presented a peak in October and November although the number of collected specimens remained small. During this period, more *An. atroparvus *were captured at Carbonnière and particularly in one horse shelter. In 1943, Sautet had observed that this species was very abundant in September suggesting a near disappearance of this species since the 1950's [9].

*Anopheles hyrcanus *presented a huge anthropophily in both areas with spectacular aggressiveness on humans: during this study scientists underwent massive attacks from females of this species. Results from others authors confirm the high level of anthropophily in France in the past or in South Eastern Europe and South Western Asia [20,21,27,29]. It was reported as one of the principal mosquito pests in Northern Greece in 2001 [29]. Unfortunately, only nine *An. hyrcanus *bloodmeals were processed because very few blood-fed *An. hyrcanus *females were captured in light traps. None of these nine blood meals were human demonstrating an opportunistic trophic behaviour of this species.

In contrast to *An. hyrcanus*, *An. melanoon *only exceptionally bite humans. This is confirmed by blood meal analyses: *An. melanoon *bite animals, especially big mammals (horses and cows). The high degree of zoophily had already been reported [15,22,27,30].

Parity rates were difficult to assess because sampling methods did not always provide enough mosquitoes suitable for dissection (too few or too dry mosquitoes). When it was possible to determine parity rates based on a robust number of specimens and using the same sampling method (light traps), parity rates were reported as inversely connected to population dynamics. This situation was observed at Marais du Vigueirat for *An. hyrcanus *(June, July and September), *An. melanoon *(July and September) and at Carbonnière for *An. hyrcanus *(July and September). Low parity rates, related to young populations, reflect the growth of populations during summer to reach a peak in August, while higher parity rates in September were related to older and decreasing populations.

## Conclusion

In the Camargue, a former malaria-endemic area, *An. hyrcanus *seems to be the only *Culicidae *likely to play a role in malaria transmission in view of its abundance and anthropophily. However, the experimental susceptibility of *An. hyrcanus *to infection with *P. vivax *and *P. falciparum *strains should be tested for assessing risks for potential transmission of this anthropophilic species. Finally, the «anophelism without malaria» situation is still going on in metropolitan France since malaria elimination, although there is a possibility for local transmission.

## Authors' contributions

NP designed the study, carried out field work, analysed data and drafted the manuscript. CT participated to field work and carried out molecular studies. GLA participated to field work. GLG participated to the design of the study and to the field work. CB participated to molecular studies. FS participated to the conception and to the design of this study. DF conceived of the study, participated in its design and coordination and helped to draft the manuscript. All authors read and approved the final manuscript.
